# Differentiation of mild cognitive impairment using an entorhinal cortex-based test of virtual reality navigation

**DOI:** 10.1093/brain/awz116

**Published:** 2019-05-28

**Authors:** David Howett, Andrea Castegnaro, Katarzyna Krzywicka, Johanna Hagman, Deepti Marchment, Richard Henson, Miguel Rio, John A King, Neil Burgess, Dennis Chan

**Affiliations:** 1 Department of Clinical Neurosciences, University of Cambridge, Cambridge, UK; 2 Institute of Cognitive Neuroscience, University College London, London, UK; 3 Department of Electrical Engineering, University College London, London, UK; 4 MRC Cognition and Brain Sciences Unit, and Department of Psychiatry, University of Cambridge, UK; 5 Department of Clinical, Educational and Health Psychology, University College London, London, UK

**Keywords:** Alzheimer’s disease, mild cognitive impairment, entorhinal cortex, path integration, virtual reality

## Abstract

The entorhinal cortex is one of the first regions to exhibit neurodegeneration in Alzheimer’s disease, and as such identification of entorhinal cortex dysfunction may aid detection of the disease in its earliest stages. Extensive evidence demonstrates that the entorhinal cortex is critically implicated in navigation underpinned by the firing of spatially modulated neurons. This study tested the hypothesis that entorhinal-based navigation is impaired in pre-dementia Alzheimer’s disease. Forty-five patients with mild cognitive impairment (26 with CSF Alzheimer’s disease biomarker data: 12 biomarker-positive and 14 biomarker-negative) and 41 healthy control participants undertook an immersive virtual reality path integration test, as a measure of entorhinal-based navigation. Behavioural performance was correlated with MRI measures of entorhinal cortex volume, and the classification accuracy of the path integration task was compared with a battery of cognitive tests considered sensitive and specific for early Alzheimer’s disease. Biomarker-positive patients exhibited larger errors in the navigation task than biomarker-negative patients, whose performance did not significantly differ from controls participants. Path-integration performance correlated with Alzheimer’s disease molecular pathology, with levels of CSF amyloid-β and total tau contributing independently to distance error. Path integration errors were negatively correlated with the volumes of the total entorhinal cortex and of its posteromedial subdivision. The path integration task demonstrated higher diagnostic sensitivity and specificity for differentiating biomarker positive versus negative patients (area under the curve = 0.90) than was achieved by the best of the cognitive tests (area under the curve = 0.57). This study demonstrates that an entorhinal cortex-based virtual reality navigation task can differentiate patients with mild cognitive impairment at low and high risk of developing dementia, with classification accuracy superior to reference cognitive tests considered to be highly sensitive to early Alzheimer’s disease. This study provides evidence that navigation tasks may aid early diagnosis of Alzheimer’s disease, and the basis of this in animal cellular and behavioural studies provides the opportunity to answer the unmet need for translatable outcome measures for comparing treatment effect across preclinical and clinical trial phases of future anti-Alzheimer’s drugs.

## Teaser

See Maass and Shine (doi:10.1093/brain/awz121) for a scientific commentary on this article.

Entorhinal cortex is affected early in Alzheimer’s disease and is critical for navigation. Using immersive virtual reality, Howett et al. reveal navigational deficits in biomarker-positive patients with mild cognitive impairment. Navigational deficits are more sensitive and specific to Alzheimer’s disease risk than a battery of reference cognitive tests.

## Introduction

To date, all interventional trials aimed at slowing the progression of Alzheimer’s disease have failed. Two of the main contributors to this failure are: (i) problems in identifying the initial stages of Alzheimer’s disease, such that interventional trials are applied too late in the disease process; and (ii) the lack of translatable outcome measures for comparing treatment effects across preclinical testing in animal models of disease and clinical trials in patient populations (Mehta *et al.*, 2017).

Detection of Alzheimer’s disease-related changes in entorhinal cortex (EC) function provides a potential solution to both of these problems. Degeneration of the EC is a critical initial stage of typical Alzheimer’s disease (Braak and Del Tredici, 2015), with 60% loss of layer II EC neurons observed by the time cognitive impairment is manifest (Gomez-Isla *et al.*, 1997). Additionally, there is emerging evidence that the initial stages of Alzheimer’s disease may be associated with the trans-neuronal spread of pathological tau within the EC-hippocampal circuit (de Calignon *et al.*, 2012; Ahmed *et al.*, 2014), prior to neocortical infiltration. As such, tests sensitive to EC function might have added value in identifying the very earliest stages of Alzheimer’s disease, prior to hippocampal involvement.

Extensive evidence from animal studies indicates that the EC is involved in spatial navigation. *In vivo* single cell studies have identified EC neurons with spatially-modulated firing patterns, including grid cells (Hafting *et al.*, 2005), head direction cells (Sargolini *et al.*, 2006) and border cells (Solstad *et al.*, 2008), with firing activity coupled to spatial behaviours (McNaughton *et al.*, 2006). Together with hippocampal place cells (O’Keefe and Dostrovsky, 1971), these EC cells are considered to represent the neural basis of a cognitive map (O’Keefe and Nadel, 1978) that mediates spatial behaviours (Fyhn *et al.*, 2007; Lester *et al.*, 2017). Within the EC, the medial EC (mEC) is considered to be particularly involved in navigation, given that up to 95% of mEC neurons may be grid cells (Diehl *et al.*, 2017), in contrast to lateral EC neurons, which exhibit little spatial selectivity. Evidence that the EC underpins navigation in other mammalian species is supported by the demonstration of EC grid cells in bats (Yartsev *et al.*, 2011), monkeys (Killian *et al.*, 2012) and humans (Jacobs *et al.*, 2013).

The EC is a phylogenetically conserved structure with distinct medial and lateral subdivisions that are homologous with the anterolateral EC (alEC) and posteromedial EC (pmEC) in humans (Navarro Schröder *et al.*, 2015; Naumann *et al.*, 2016, 2018). Sensory characteristics of objects appear to be represented in the alEC (Olsen *et al.*, 2017; Yeung *et al.*, 2017; Reagh *et al.*, 2018) while representations of scenes and current location based upon self-motion are represented in the pmEC (Reagh and Yassa, 2014; Berron *et al.*, 2018; Campbell *et al.*, 2018), with both streams converging on the hippocampus. Thus, integration of self-motion cues requires the pmEC, whereas remembering the spatial configuration of an environment and the objects/contents within it is dependent upon the hippocampus (Knierim *et al.*, 2014). Path integration is the ability to keep track of, and return to, a previously visited location and is dependent upon the continuous integration of multisensory cues (visual, proprioceptive and vestibular) representing current position and heading direction in reference to a fixed location (Etienne and Jeffery, 2004; McNaughton *et al.*, 2006). While several brain regions are thought to contribute to path integration there is robust evidence that the EC and the periodic firing of grid cells are central to this navigation strategy (McNaughton *et al.*, 2006; Burgess, 2008), supplemented by inputs from other EC cells with spatially-modulated firing, notably head direction cells. Moreover in rodents, path integration deficits are elicited by mEC lesions (Parron and Save, 2004; Van Cauter *et al.*, 2013; Knierim *et al.*, 2014; Jacob *et al.*, 2017) and attenuated grid cell firing achieved via both mEC layer 2 knockout (Tennant *et al.*, 2018), and glutamatergic receptor 1 knockout (Allen *et al.*, 2014). This evidence from the animal literature is reinforced by human imaging studies that demonstrate the role of the EC in components of path integration such as route planning (Maguire *et al.*, 1998; Jacobs *et al.*, 2010), computation of goal direction (Chadwick *et al.*, 2015) and goal distance (Spiers and Maguire, 2007; Howard *et al.*, 2014). Finally, dysfunction of grid cell-like activity is associated with path integration deficits in older adults (Stangl *et al.*, 2018).

Spatial tests, based on the cognitive map theory, have already shown that spatial processing is impaired in early Alzheimer’s disease. The Four Mountains Test (4MT), a hippocampal-dependent test of allocentric spatial memory (Hartley *et al.*, 2007), differentiates patients with mild cognitive impairment (MCI) with and without CSF biomarkers of Alzheimer’s disease (MCI+ and MCI−, respectively) (Moodley *et al.*, 2015) and is predictive of conversion from MCI to dementia (Wood *et al.*, 2016). Crucially for detection of Alzheimer’s disease prior to symptom onset, performance on the 4MT correlates with dementia risk score in asymptomatic 40–59 year olds (Ritchie *et al.*, 2018), while young adult *APOE* ɛ4 carriers at increased risk of Alzheimer’s disease exhibit reduced grid-cell like representations and activation of the EC during a functional MRI navigation task (Kunz *et al.*, 2015). Finally, impaired route learning and way-finding is observed in asymptomatic individuals with positive amyloid-PET scans (Allison *et al.*, 2016).

**Figure 1:**
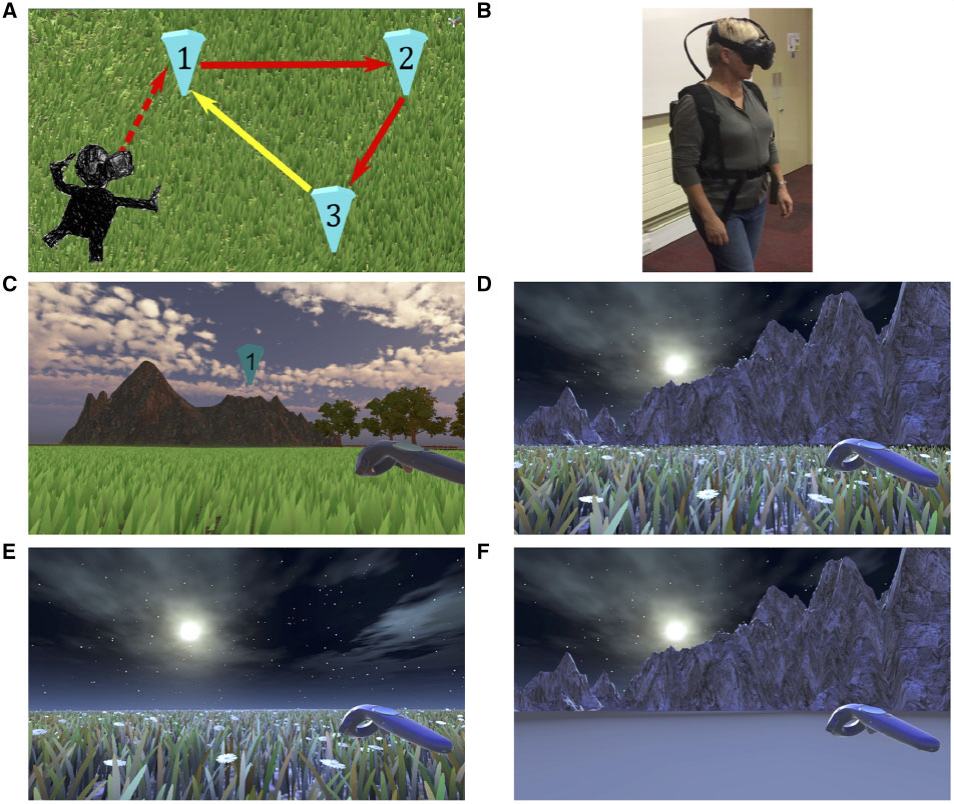
**Path integration task.** (**A**) Illustration of the path integration task. Each numbered inverted blue cone is a location marker. Only one cone was visible at a time; upon reaching a blue cone it disappeared and the next one in the sequence appeared. Red arrows indicate the guided sequence along two sides of the triangle. The yellow arrow, the last side of the triangle, signifies the assessed return path, performed in the absence of any cones. (**B**) Demonstration of VR equipment on a participant during the task, used with permission. (**C**) Example environment from the head mounted display with textural and boundary cues present, with cone 1 and the controller shown. Texture and boundary cues are present in all trials when navigating between cones. (**D**–**F**) Return conditions applied when attempting to return to the location of cone 1 only (yellow arrow, **A**) and included no change (**D**), removal of environment boundaries (**E**) and removal of surface detail (**F**).

These previous studies provide the backdrop for the present study, which investigates EC-based navigation in MCI patients at risk of developing dementia. Navigation will be tested using a path integration task (Fig. 1A). In this study, path integration will be tested using an immersive virtual reality (iVR) paradigm where participants navigate by real-world walking within simulated environments. Immersive VR has several theoretical and operational advantages over ‘desktop’ VR tasks, which are typically performed seated and thus without locomotor or proprioceptive feedback, both of which are pivotal for grid cell function (Winter *et al.*, 2015). First, the actual movement in iVR approximates real world navigation and thus has greater ecological validity than desktop VR. Second, there is evidence of differing neural processes underlying desktop and actual navigation, with desktop VR being associated with lower frequency hippocampal theta oscillations (Bohbot *et al.*, 2017). This has negative implications for desktop VR as a valid proxy for real life navigation. Lastly, larger rotational (Klatzky *et al.*, 1998) and distance errors (Distler *et al.*, 1998; Sinai *et al.*, 1999; Adamo *et al.*, 2012) have been reported in desktop VR navigation when compared with tasks requiring active movement, possibly reflecting the absence of self-motion cues, leading in turn to reduced grid cell activation (Ólafsdóttir and Barry, 2015).

The primary objective of this study was to test the hypothesis that performance on an iVR path integration task of EC function would differentiate MCI patients at increased risk of developing dementia. The secondary study objectives were to determine: (i) whether manipulation of the environmental conditions would affect path integration performance; (ii) whether path integration test performance correlates with EC volumes; and (iii) whether the path integration task exhibits greater classification accuracy than current cognitive tests considered to have high diagnostic sensitivity and specificity for early Alzheimer’s disease.

## Materials and methods

### Participants

Patients with MCI (*n = *45) were recruited from the Cambridge University Hospitals NHS Trust Mild Cognitive Impairment and Memory Clinics. MCI was diagnosed by neurologists according to the Petersen criteria (Petersen, 2004), diagnosis of which requires: (i) subjective cognitive complaint; (ii) objective evidence of cognitive impairment; (iii) preserved activities of daily living; (iv) functional independence; and (v) absence of dementia. Objective cognitive decline was evaluated using the Addenbrooke’s Cognitive Examination-Revised (ACE-R; Mioshi *et al.*, 2006) and a score of 0.5 on the Clinical Dementia Rating scale (CDR) (Morris, 1997). All patients underwent screening blood tests to exclude reversible causes of cognitive impairment. Exclusion criteria included the presence of a major medical or psychiatric disorder, epilepsy, a Hachinski Ischaemic Score >4 (Moroney *et al.*, 1997), a history of alcohol excess or any visual or mobility impairment of such severity as to compromise ability to undertake the iVR test.

Twenty-six patients with MCI underwent CSF biomarker studies (amyloid-β_1–42_, total tau, phosphorylated tau) as part of their clinical diagnostic workup. Biomarker studies were undertaken using ELISA assay kits (Innotest, Innogenetics) as outlined elsewhere (Shaw *et al.*, 2009). Thresholds for positivity were set as CSF amyloid <550 pg/ml, CSF tau >375 pg/ml with a CSF tau: amyloid ratio of >0.8 (Mulder *et al.*, 2010). MCI patients were stratified into biomarker-positive (MCI+, *n = *12) and biomarker-negative (MCI−, *n = *14) groups (Table 1). Researchers undertaking the VR tests were blinded to the CSF status of patients. The remaining 19 patients with MCI did not undergo CSF studies as part of their clinical workup. Healthy control participants without a history of cognitive impairment (healthy control subjects, *n = *41, Table 1) were recruited from Join Dementia Research, an online repository of patients and volunteers interested in participating in dementia research.

**Table 1:** Demographics and neuropsychological test scores of across patients with mild cognitive impairment and healthy controls participants

	**Healthy controls (*n = *41)**	**MCI (*n = *45)**		
		**MCI (*n = *45)**	**Negative (*n = *14)**	**Positive (*n = *12)**
Age	69.3 ± 7.5	71.7 ± 8.3 ^n.s^	71.1 ± 9.0	75.4 ± 7.0 ^n.s^
Males (%)	15 (36)	12 (63)^n.s^	10 (71)	9 (75)^n.s^
Years in education	14.8 ± 3.61	14.2 ± 3.37 ^n.s^	14.5 ± 4.4	14.5 ± 3.8 ^n.s^
ACE-R	97.2 ± 3.2	89.3 ± 5.4*	86.6 ± 7.6	80.1 ± 12.1 ^n.s^
MMSE	29.7 ± 0.6	27.90 ± 1.7*	27.6 ± 2.6	25.0 ± 1.7 ^n.s^
NART Errors	6.28 ± 3.40	17 ± 10.95*	13.1 ± 8.9	9.1 ± 6.8 ^n.s^
Rey Figure Recall				
Copy	36 ± 0	34.2 ± 2.7*	34.4 ± 1.7	33.1 ± 4.4 ^n.s^
Immediate	22.2 ± 7.6	17.5 ± 9.8*	13.8 ± 8.3	9.6 ± 9.1 ^n.s^
Delayed	21.3 ± 7.9	15.8 ± 11.0*	12.8 ± 9.8	8.3 ± 9.6 ^n.s^
FCSRT immediate				
Free	34.3 ± 5.1	24.9 ± 11.5*	22.1 ± 9.2	15.4 ± 11.4*
Total	47.6 ± 0.6	44.7 ± 5.7*	43.1 ± 8.3	36.1 ± 11.5 ^n.s^
FCSRT delayed				
Free	13.4 ± 1.5	9.3 ± 5.3*	7.9 ± 5.1	4.8 ± 4.8 ^n.s^
Total	16 ± 0	14.8 ± 2.3*	13.9 ± 4.1	12.3 ± 4.0 ^n.s^
Trails B, s	77.2 ± 26.5	145.6 ± 72.8*	130.1 ± 42.2	152.6 ± 88.6 ^n.s^
Digit Symbol	64.2 ± 14.5	49.9 ± 14.1*	47.0 ± 7.5	43.7 ± 13.7 ^n.s^
4MT	10.8 ± 1.8	9.3 ± 3.0*	7.3 ± 3.4	6.8 ± 2.2 ^n.s^

Between group differences in neuropsychological test performance were assessed between healthy control subjects versus MCI as a whole, and MCI+ versus MCI−, scores indicate number of correct responses unless otherwise indicated. *P < 0.05, *P < 0.02 (Bonferroni-adjusted alpha); n.s = *P > *0.05.

Digit Symbol = Digit Symbol Substitution Test; FCSRT = Free and Cued Selective Reminding Test; MMSE = Mini-Mental State Examination; NART = National Adult Reading Test; Trails B = Trail Making Test B.

The study was undertaken in line with the regulations outlined in the Declaration of Helsinki (WMA, 2013) and was approved by the NHS Cambridge South Research Ethics Committee (REC reference: 16/EE/0215).

### The immersive virtual reality path integration task

The path integration task was administered using the HTC Vive iVR kit (Fig. 1B), which uses external base stations to map out a 3.5 × 3.5 m space within which participants walked during the VR task. If participants went beyond the tracked boundary by 30 cm, an ‘out of border’ warning appeared in their sightline to encourage them to not walk any further. Researchers were also in the immediate proximity to ensure that participants did not venture beyond the test space.

The task was programmed in the Unity game engine and ran on Steam VR software, running on an MSI VR One backpack laptop.

The path integration task was undertaken within virtual open arena environments with boundary cues projected to infinity (Fig. 1C). Three environments were used, each with unique surface details, boundary cues and lighting. The absence of local landmarks ensured EC-grid cell dependent strategies rather than striatal-mediated landmark-based navigation (Doeller *et al.*, 2008).

No enclosure or local landmarks were present during the task in order to exclude any non-path integration compensatory navigation strategies. A 1:1 correspondence between movement in the real and virtual worlds eliminated vestibular mismatch and minimized nausea and other tolerability issues.

Participants were asked to walk an ‘L’-shaped outward path to three different locations, each marked by inverted cones at head height numbered one, two and three (Fig. 1A and C). Only one cone was present at a time, each cone would disappear after the participant reached it and the next cone in sequence would appear. An auditory stimulus was presented with a cone’s appearance prompting participants toward the next cone’s location. Upon reaching cone 3, a message projected onto the virtual scene asking participants to walk back to their remembered location of cone 1 (return path). When they reached their estimated location of cone 1, participants pressed a trigger on a hand-held controller that logged their location and ended the trial.

Pre-trial practice sessions consisted of 20 s of habituation to the iVR environment, during which participants were encouraged to explore the environment. Following habituation, participants performed five practice trials, where cone 1 was re-presented at the end of each trial to provide direct visual feedback to participants on the distance error between the remembered and actual locations of cone 1.

The task consisted of nine trials conducted within each of the three environments, totalling 27 trials per participant. To examine the effects of environmental cues on path integration, the environment was altered during the return path when participants were attempting to return to the remembered location of cone 1. Three return conditions were used: condition A, no environmental change (Fig. 1D); condition B, removal of boundary cues (Fig. 1E); and condition C, removal of surface detail (Fig. 1F).

Each return condition was presented three times per environment, with return conditions presented pseudo-randomly in each environment in order to ensure participants were relying more on proprioceptive and self-motion cues rather than allothetic strategies.

Condition B was designed to increase dependence on self-motion cues and homing vector calculation by removing boundary cue information (Burgess *et al.*, 2004), thereby placing a greater cognitive load on the path integration network (Zhao and Warren, 2015). Condition C was designed to prevent feedback from surface motion during locomotion, thereby disrupting optic flow (Kearns *et al.*, 2002) and increasing dependence on allocentric representations of space (Nardini *et al.*, 2008). As such, return conditions B and C were considered analogous to ‘stress tests’ for EC network-dependent navigation, with the prediction that a greater impairment in task performance would be observed during these conditions, compared with condition A.

**Figure 2:**
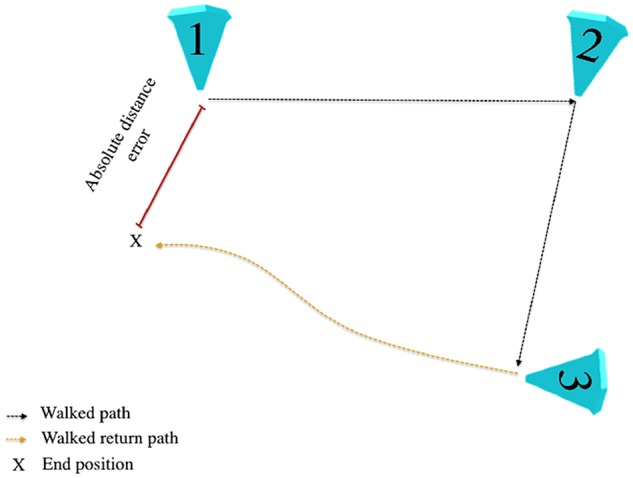
**Primary measures of performance accuracy.** Absolute distance error is defined as the Euclidean distance between the participant’s estimate of location one (goal) and the actual location of cone 1.

Performance in the iVR path integration task was assessed using three outcome measures. Absolute distance error, the primary outcome measure, was defined as the Euclidean distance between estimated and actual location of cone 1, in line with previous research (Fig. 2; Chrastil *et al.*, 2015; Mokrisova *et al.*, 2016). Two secondary outcome measures were included to deconstruct absolute distance errors into its proportional angular and linear components. These measures additionally controlled for between-trial variance in triangle geometry owing to the pseudo-random generation of cone locations that could affect task difficulty, although variance is minimal in paths <10 m long (Harris and Wolbers, 2012). Proportional angular errors reflect the accuracy of performed rotation at cone 3 toward the participant’s estimated location of cone 1 compared to the optimal rotation required to align with cone 1 (Supplementary Fig. 1A). Proportional linear errors reflected the accuracy of distance estimation, with the Euclidean distance travelled between cone 3 and the participant’s estimated location of cone 1 compared to the distance between cone 3 and actual location of cone 1 (Supplementary Fig. 1B).

### MRI acquisition and analysis

Thirty-seven healthy control subjects and 34 MCI patients (11 MCI+, 9 MCI−) underwent MRI scanning on 32 channel Siemens 3 T Prisma scanners based either at the MRC Cognition and Brain Sciences Unit, Cambridge, or the Wolfson Brain Imaging Centre, Cambridge, with the same acquisition parameters used at the two scan sites. The scan protocol included whole brain 1 × 1 × 1 mm T_1_-weighted MPRAGE (acquisition time 5 min 12 s, repetition time 2300 ms, echo time 2.96 ms) and high-resolution 0.4 × 0.4 × 2 mm T_2_-weighted scans through the hippocampal formation with scans aligned orthogonally to the long axis of the hippocampus (acquisition time 8 min 11 s, repetition time 8020 ms, echo time 50 ms).

**Figure 3:**
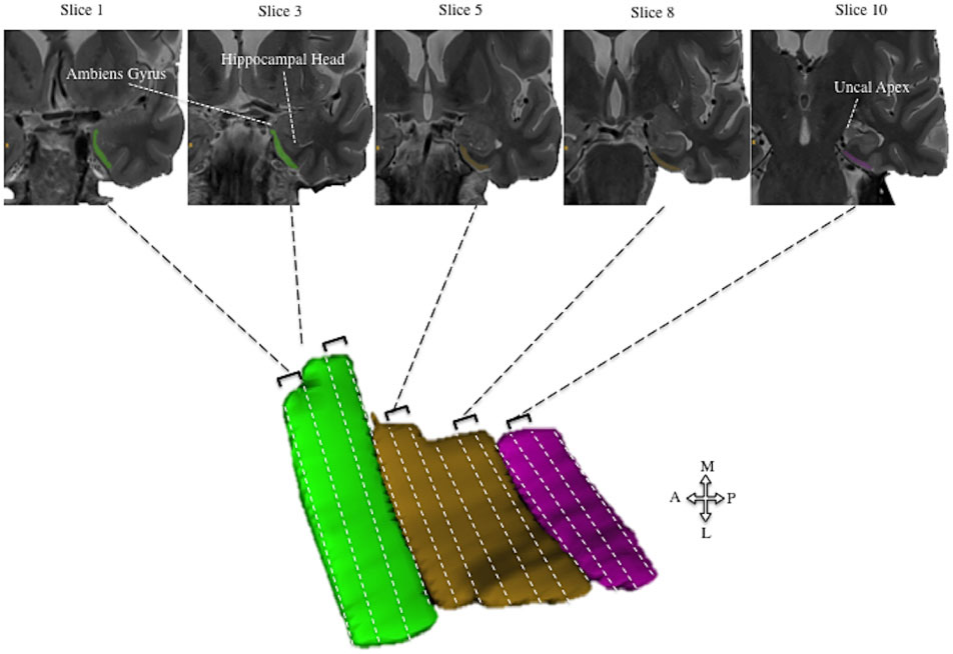
**Protocol for the segmentation of the whole entorhinal cortex and partial segmentation of its anteriolateral and posterioromedial subdivisions.** Anteriolateral EC (alEC, green) is segmented two slices anterior to the emergence of the hippocampal head (slice 3). Posteromedial (pmEC, pink) is segmented from one slice anterior to, and one slice posterior from, the uncal apex (slice 10). This partial approach for EC subdivisions does not encompasses the full extent of the EC but rather reflects the anterior and posterior extremes of the EC that avoid segmenting the progressive boundary in the absence of consistent landmarks. Correspondingly, all intermediate slices between alEC and pmEC are segmented as EC (brown), total EC volume is produced by summing all three EC subdivision volumes. Arrow schematic indicates anatomical plane for 3D segmentation.

As well as segmentation of the whole EC, additional segmentation of the alEC and pmEC subregions, representing the human homologues of the rodent lateral and medial EC, respectively, was undertaken given their differing roles in spatial processing (Van Cauter *et al.*, 2013; Knierim *et al.*, 2014). Converging evidence from functional MRI studies indicate a functional domain-specificity to the EC that mirrors rodent research, whereby the pmEC and alEC is implicated in processing scene and object information, respectively (Maass *et al.*, 2015; Navarro Schröder *et al.*, 2015). Whilst age-related deficits in object-related processing have been related to alEC hypoactivity (Berron *et al.*, 2018; Reagh *et al.*, 2018), no research to date has investigated the relationship between EC subfield volumetry and performance in navigational tasks in either MCI or Alzheimer’s disease. While segmentation protocols for these subregions are available at 7 T (Maass *et al.*, 2015), no complete protocol is available for segmentation at 3 T. Therefore, for this study, an in-house protocol was devised that partially segmented alEC and pmEC using the three anterior-most and three posterior-most slices of the EC. Intermediate slices were not used for alEC and pmEC segmentation because of the overlap of the two subdivisions within this part of the EC and the absence of robust anatomical landmarks for delineating this progressive boundary. As such, this protocol prioritized specificity of segmentation over completeness (Supplementary material). Manual segmentation was performed in ITK-SNAP (Yushkevich *et al.*, 2006) (Fig. 3, detailed protocol in Supplementary material). High inter- and intra- rater reliability was achieved for the manual segmentation protocol of the EC, alEC and pmEC, consistent with previous research (Berron *et al.*, 2017; Olsen *et al.*, 2017) (Supplementary Table 1).

Given the involvement of the hippocampus and retrosplenial cortex in path integration (Worsley *et al.*, 2001; Chrastil *et al.*, 2015) these additional regions of interest were also segmented using Freesurfer 6.0 (Fischl *et al.*, 2002; Iglesias *et al.*, 2015). In the absence of an automated protocol for the complete segmentation of the retrosplenial cortex, posterior cingulate cortex (PCC) and isthmus of cingulate cortex masks were used as a proxy measure of the retrosplenial cortex, with the former encompasses the retrosplenial cortex along with other structures and the latter targeting the ventral retrosplenial cortex implicated in processing scene information (Vann *et al.*, 2009; Mitchell *et al.*, 2018).

All segmentations were manually inspected to exclude cysts, CSF and meninges; all volumetric measurements were averaged between hemispheres and normalized to intracranial volume.

### Comparator neuropsychological tests

To compare the ability of the iVR test to classify prodromal Alzheimer’s disease with that of reference neuropsychological tests considered to be highly sensitive to early Alzheimer’s disease. All participants were administered a battery of tests chosen for their effectiveness in predicting conversion from MCI to dementia (i–iii), inclusion in the Preclinical Alzheimer’s Cognitive Composite (i and iv) approved by the FDA for use as cognitive outcome measures in trials aimed at preclinical Alzheimer’s disease, or prior work indicating high sensitivity and specificity for prodromal Alzheimer’s disease (v).

These tests are as follows (cognitive domains assessed in parentheses): (i) Free and Cued Selective Reminding Test (FCSRT, episodic memory – verbal; Buschke, 1984); (ii) Rey figure recall (RFR, episodic memory – non-verbal, Osterrieth, 1944); (iii) Trail Making Test B (TMT-B; executive function, attention, processing speed; Bowie and Harvey, 2006); (iv) Digit Symbol test (DST, attention, processing speed, Ryan and Lopez, 2001); and (v) 4MT (allocentric spatial memory; Hartley *et al.*, 2007). All participants also underwent global cognitive testing with the ACE-R (Mioshi *et al.*, 2006) and the National Adult Reading Test (NART; Nelson, 1982), as a measure of premorbid IQ.

### Statistical analysis

Demographic differences between MCI+ and MCI− were assessed using one-way ANOVA, or the Kruskal Wallis test where parametric assumptions were violated, whereas differences between healthy control subjects and total (combined) MCI were assessed using *t*-tests or non-parametric Mann-Whitney test.

Between-group performance in the path integration task compared all MCIs against healthy control subjects, as well as MCI+ against MCI−. Linear mixed effect modelling (LME) was used to assess the effect of MCI status on absolute distance error, proportional angular error and proportional linear error. LMEs are the most suitable method for analysing clustered datasets (27 trials with one of three return conditions per trial per participant), with missing data (excluded due to travelling ‘out of border’, see ‘Results’ section), and unbalanced designs (Moen *et al.*, 2016). Final model fixed effects included an interaction term between diagnosis and return condition, along with covariates of age, sex, years in education, ACE-R, NART and VR environment. Unique participant identifiers were used as the random intercept and VR environment as random coefficient, for further details see the Supplementary material. Reported denominator degrees of freedom were computed using the conservative Satterthwaite approximation.

Between-group differences in region of interest volumetry and cognitive performance across the neuropsychological test battery were investigated using one-way ANCOVA—rank ordered where parametric assumptions were violated (Conover and Iman, 1982)—covarying for age, sex and years in education. Separate linear regression models were used to assess absolute distance error (averaged per participant across all trials) and region of interest volumetry, adjusting for age, sex, years in education and mean path integration performance per participant group. Analyses were conducted across all participants and between MCI+ versus MCI−. Bonferroni correction was used to control for planned multiple comparisons. Residuals were visually inspected for violating linear assumptions, leverage and outliers.

The classification ability (MCI from healthy control subjects or MCI+ from MCI−) of the path integration test (using per-participant averages of each path integration outcome measure) was compared to reference cognitive tests. All linear classification models were adjusted for age, sex and years in education and used k-fold cross-validation (k = 10) to control for over-fitting (Hawkins *et al.*, 2003). Posterior probabilities following cross-validation were used to generate area under the curve (AUC) of the receiver operating characteristic (ROC), as well as optimal sensitivity and specificity. Pointwise confidence intervals were generated following bootstrapping with 1000 replications.

All analysis was conducted in MATLAB 2017b (Mathworks, https://uk.mathworks.com/).

### Data availability

Anonymized data are available on request.

## Results

### Demographics and neuropsychological testing

No significant differences in age, gender, or years in education were observed between all MCI and healthy control subjects, or between MCI+ and MCI− (Table 1). Following Bonferroni correction with an adjusted α of 0.002, the MCI group as a whole exhibited significantly more errors in all neuropsychological tests compared to healthy control subjects (*P* < 0.002), whereas no difference between MCI+ and MCI− survived multiple comparisons (*P* > 0.002).

### Immersive virtual reality path integration task

Of 2295 trials, 775 were excluded (33.77%) because of the ‘out of border’ boundary being reached during the return path, leaving 1520 viable trial remaining for analysis, with no between group difference in ‘out of border’ warnings were observed (*P > *0.05). All participants successfully completed the path integration task with no reported nausea or tolerability issues.

### Absolute distance error

**Figure 4:**
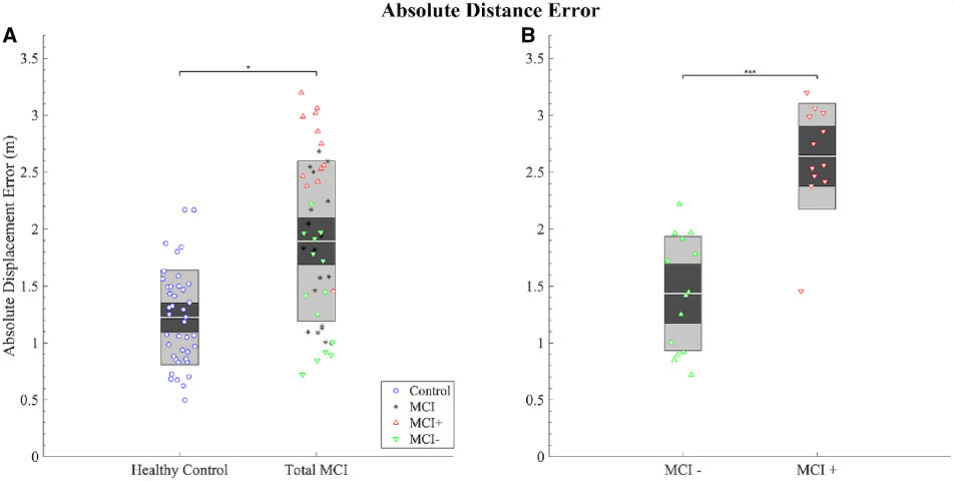
**Graph summarizing the between group differences in path integration performance.** Absolute distance error (Euclidean distance) error in metres (**A** and **B**). (**A**) Group comparison between healthy controls and total MCI and (**B**) between MCI− and MCI+. Each marker represents the mean performance across trials of each individual: blue circles = healthy control subjects; black asterisks = MCI without biomarkers; red triangles = MCI+; green inverted triangles = MCI−; central grey line = mean; dark grey inner box = 95% CIs; light grey outer box = 1 standard deviation. *P < 0.05, ***P < 0.001.

The MCI group as a whole exhibited significantly larger absolute distance errors than the healthy control group [*t*(1,107) = 3.24, *P* < 0.01, Fig. 4A], with an estimated 57.33 ± 17.87 cm increase in absolute distance error compared to healthy control subjects. MCI+ patients exhibited significantly larger absolute distance errors compared MCI− patients [*t*(1,163) = 4.69, *P < *0.001, Fig. 4B], with an estimated increase of 97.56 ± 20.34 cm compared to MCI−. ACE-R score correlated with absolute distance errors across healthy control subjects and total MCI patients [*t*(1,85) = 2.89, *P < *0.01] and across MCI+ and MCI− groups [*t*(1,26) = 4.01, *P < *0.01], with lower ACE-R scores being associated with greater distance errors.

To assess whether tau and amyloid-β contributed toward absolute distance error a mixed effect model was performed with both CSF measures z-scored, controlling for years in education, sex and age. It was found that the model explained 64% of the variance (R^2^) with CSF biomarkers being significant predictors of absolute distance error. Absolute distance error was positively correlated with CSF total tau [*t*(1,25) = 2.18, *P < *0.05] and negatively associated with CSF amyloid-β [*t*(1,25) = −4.39, *P < *0.001; Supplementary Fig. 2].

Neither sex, years in education or age were predictive of absolute distance error (*P > *0.05). An interaction between CSF tau and CSF amyloid-β was also examined but this addition neither improved model fit, as indicated by ratio likelihood testing, or was significant, and thus was not used in the final analysis.

Similar main effects of diagnosis were observed in proportional linear errors between healthy control subjects and total MCI group [*t*(1,95) = 2.27, *P < *0.05], as well as between MCI+ and MCI− groups [*t*(1,87) = 3.09, *P < *0.001], but were not observed in proportional angular errors (*P > *0.05 across both groups; see Supplementary material). No other fixed effect was associated with any of the outcome measures of the path integration task.

### Effect of return condition on path integration

**Figure 5:**
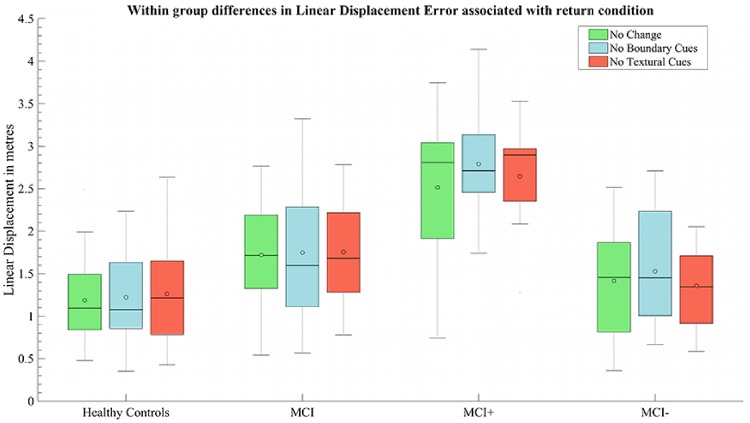
**The effect of return condition within participant groups.** The effect of return condition on absolute distance error averaged per participant in each group. Return conditions: green = no environmental change; blue = removal of distal boundary cues; red = removal of surface detail; open circle = mean; black line = median.

No main effects of return condition was observed on absolute distance error between healthy control and total MCI groups [*F*(2,1318) = 0.86, *P* > 0.05; Fig. 5] or between MCI+ and MCI− groups [*F*(2,384) = 0.56, *P > *0.05]. Interaction terms between return condition and participant grouping were also included in the analyses to examine the differential influence of return conditions on path integration performance for MCI+ (compared to MCI−) and MCI as a whole (compared to healthy control subjects). However, no interaction was observed between healthy control and total MCI groups [*F*(2,1325) = 0.83, *P* > 0.05] or between MCI+ and MCI− groups [*F*(2,386) = 0.55, *P > *0.05].

For proportional angular errors, a trend toward an interaction between biomarker status (MCI+ versus MCI−) and return conditions B and C was observed [*F*(2,398) = 2.93, *P < *0.05; Supplementary Fig. 5A], but this did not survive Bonferroni correction. No other main effect of return condition or interaction with diagnostic status was observed for proportional outcome measures (*P > *0.05, see Supplementary material).

### Group differences in MRI volumetry and association with path integration performance

Group-level analyses adjusted for age, sex and years in education, revealed reduced region of interest volumetry (PCC, hippocampus, EC, alEC and pmEC) in the total MCI group compared to healthy control subjects (*P < *0.05), and in the MCI+ group compared to the MCI− group (*P < *0.05). However, across healthy control subjects and total MCI, only hippocampal [*F*(1,66) = 13.32, *P < *0.001], EC [*F*(1,66) = 33.14, *P < *0.001], alEC [F(1,66) = 21.87, *P < *0.001] and pmEC [*F*(1,66) = 12.16, *P < *0.001] volumes survived the Bonferroni adjusted α of 0.005. No volumetric difference of the isthmus was observed across all participants or MCI+ and MCI− groups (*P > *0.05, see Supplementary Table 2 for full results).

**Figure 6:**
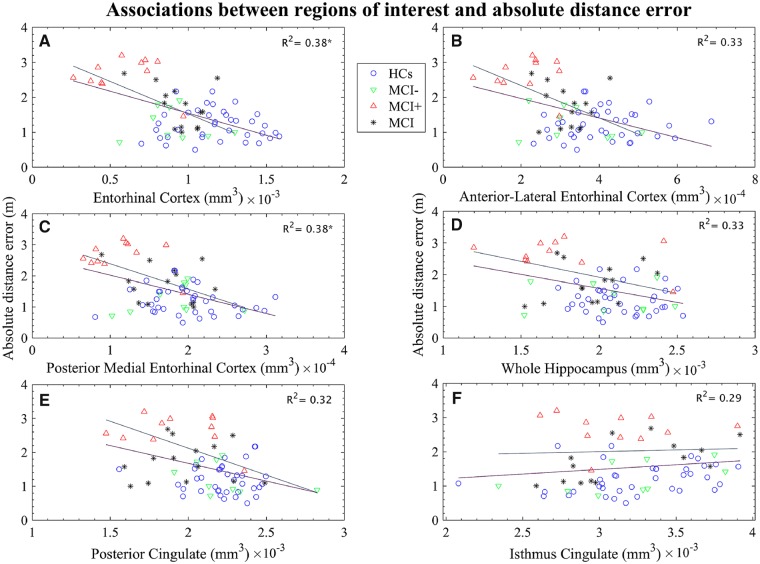
**Scatterplots of path integration performance and region of interest volumetry.** The relationship absolute distance error and regions of interest including; EC (**A**), alEC (**B**), pmEC (**C**), whole hippocampus (**D**)**,** PCC (**E**) and isthus of the cingulate cortex (**F**) was assessed. Least square lines are group specific: grey = across MCI+and MCI−; purple = all participants), *P < 0.005 (Bonferroni adjusted α).

Associations between regions of interest and path integration performance were assessed across total MCI and healthy control groups (Fig 6, purple line) and across MCI+ and MCI− groups (Fig. 6, grey line), adjusting for age, sex, years in education and average path integration performance per participant group. Significant negative associations, surviving the Bonferroni adjusted α of 0.005, were observed across all participants between absolute distance errors and both total EC [*F*(1,64) = 9.60, *P < *0.005; Fig. 6A] and pmEC [*F*(1,64) = 9.73, *P < *0.005; Fig. 6C] volumes, each with an R^2^ of 0.38. Across healthy control and total MCI groups, associations between absolute distance error, alEC (*P = *0.04, Fig. 6B) and hippocampal (*P = *0.03, Fig. 6D) volumes did not survive Bonferroni correction. Similarly, for MCI+ and MCI− group comparisons, the association between hippocampal volume and absolute distance error (Fig. 6D, *P = *0.02) did not survive multiple comparison correction. Neither posterior cingulate nor isthmus cingulate volumes were significant predictors of absolute distance error across all participants (PCC: *t* = 1.95, *P > *0.05; isthmus cingulate cortex: *t* = 0.35, *P > *0.05) or MCI+ and MCI− (PCC: *t* = 1.47, *P > *0.05; isthmus cingulate cortex: *t* = 0.19, *P > *0.05).

Additional analyses used multiple linear regression to examine the strength of EC and pmEC associations with absolute distance error whilst controlling for both hippocampal and alEC volumes across all participants. EC [*F*(1,63) = 6.09, *P < *0.05) and pmEC [*F*(1,63) = 6.14, *P < *0.03] models were significant, both EC (*t* = 2.20, *P < *0.05) and pmEC (*t* = 2.23, *P < *0.05) volumes were significant predictors of absolute displacement error but neither survive multiple comparison corrections. To examine further the neural correlates of path integration performance, a hypothesis-free backward stepwise regression was performed, with absolute distance error as the response variable and regions of interest from the Desikan–Killiany–Tourville atlas (Klein and Tourville, 2012, averaged across hemispheres and normalized to intracranial volume) as predictor variables, along with patient status, age, sex and years in education. Predictor variable inclusion was determined by the Akaike information criterion and the final model’s predictor variables were refined by examination of variance inflation factors in an attempt to minimize collinearity. Finally, false discovery rate with a stringent alpha of 0.01 was used to control for multiple comparisons. The final model was significant [*F*(34,36) = 5.79, *P < *0.001], explaining 86.51% (R^2^) of the variance in absolute distance error. Following control for multiple comparisons, 20 brain volumes significantly contributed to the final model and these are summarized in Supplementary Table 3. However, stepwise regression can be affected by collinearity and as such the interpretation of these additional region of interest analyses needs to be made with caution (Thompson, 1995).

Lastly, given the demonstrated high accuracy of the 4MT in differentiating MCI due to underlying Alzheimer’s disease (Moodley *et al.*, 2015) and its predictive subsequent conversion to dementia (Wood *et al.*, 2016), an ANCOVA adjusted for age, sex, years in education and average group performance was used to examine the relationship between 4MT scores and both the pmEC and hippocampus across all participants and MCI+ and MCI−. Across all participants, the model was significant for both hippocampal [*F*(1,64) = 6.54, *P < *0.001] and pmEC volumes [*F*(1,64) = 5.63, *P < *0.001]; however, neither hippocampal (*t* = 1.92, *P > *0.05) or pmEC (*t* = 0.73, *P > *0.05) volumes were significant predictors of 4MT score. Across MCI + and MCI − neither hippocampus [*F*(1,14) = 1.36, *P < *0.05] or pmEC models were significant [*F*(1,14) = 1.37, *P > *0.05].

### Receiver operating curves curves and classification accuracy

**Figure 7:**
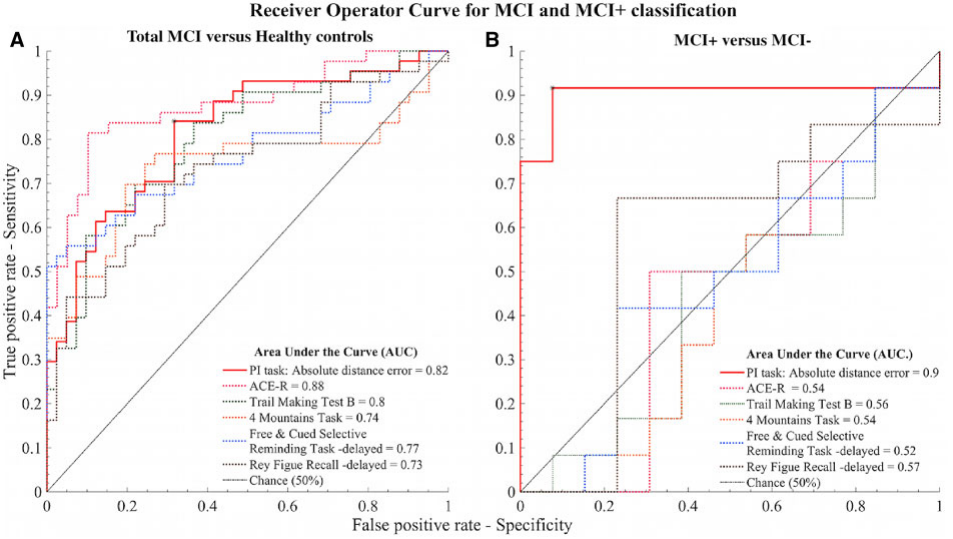
**ROC plot.** Accuracy of path integration task performance for classifying (**A**) total MCI from healthy control subjects and (**B**) MCI+ from MCI− patients. Path integration performance is represented by absolute distance error (solid red line). Classification of reference cognitive tests is represented by dashed lines for comparison. ACE-R (grey), Trail Making Test B (green), 4MT (yellow), Free and Cued Selective Reminding Test – delayed free recall (blue) and Rey Figure Recall – delayed recall (purple). Asterisk indicates optimal operating point for absolute distance error.

AUC, sensitivity and specificity were estimated using k-fold cross-validation (k = 10), adjusted for age, sex and years in education. For the classification of total MCI patients from healthy control subjects, absolute distance error was associated with an AUC of 0.82 [Fig. 7A; 95% confidence intervals (CI) = 0.71–0.89], with an error ≥ 157 cm yielding a sensitivity of 0.84 and specificity of 0.68. By comparison, the ACE-R was associated with an AUC = 0.86 (CI = 0.79–0.94), TMT-B (AUC = 0.79, CI = 0.68–0.87), 4MT (AUC = 0.73, CI = 0.6–0.83) and the delayed conditions of FCSRT (AUC = 0.73, CI = 0.61–0.85) and Rey-Osterrieth Figure Recall Test (AUC = 0.72, CI = 0.60–0.83).

Classification accuracy of MCI+ from MCI− using absolute distance error was very high, with an AUC of 0.90 (Fig 7B; CI = 0.59–1), and errors ≥ 196 cm yielding a sensitivity and specificity of 0.92 for both. This AUC was considerably higher than that of the comparator reference cognitive tests: ACE-R (AUC = 0.53, CI = 0.24–0.73), TMT-B (AUC = 0.57, CI = 0.22–0.69), 4MT (AUC = 0.56, CI = 0.22–0.72) and the delayed conditions of FCSRT (AUC = 0.57, CI = 0.22–0.68) and Rey-Osterrieth Figure Recall Test (AUC = 0.55, CI = 0.22–0.68), indicating a markedly superior ability of the path integration test to differentiate MCI+ from MCI−.

## Discussion

This study demonstrated that performance on a novel immersive virtual reality path integration paradigm, based on the central role of the entorhinal cortex in navigation, was impaired in MCI patients compared to healthy controls. In keeping with the study hypothesis that a navigation task based upon theories of EC function can differentiate MCI patients at increased risk of developing dementia, we found that Alzheimer’s disease biomarker-positive patients drove the difference in navigation accuracy between MCI patients and controls. Consistent with the postulated role of the EC in navigation, and the specific role of the pmEC subdivision in spatial processing, larger path integration performance errors were associated with smaller total EC and pmEC subdivision volumes across all participants. Finally, and of high relevance for potential diagnostic usage, path integration performance differentiated MCI biomarker-positive patients, i.e. those with prodromal Alzheimer’s disease, from biomarker-negative patients with markedly higher sensitivity and specificity than a battery of ‘gold standard’ cognitive tests used in clinical and research practice.

The navigational impairments observed in MCI patients is in line with previous navigation research (Hort *et al.*, 2007; Laczó *et al.*, 2014; Peter *et al.*, 2018) and with the sparse literature on real-space path integration in MCI and Alzheimer’s disease (Mokrisova *et al.*, 2016). Significantly larger absolute distance errors were observed in MCI+ than in MCI−, with near-total separation of these two groups on this primary outcome measure, with the latter group exhibiting comparable performance to healthy control subjects. Additional analyses revealed that both CSF total tau and CSF amyloid-β were highly predictive of absolute distance error, independent of age, sex and years in education, supporting the notion that path integration deficits are related to Alzheimer’s disease molecular pathology. Collectively, these data suggest that navigational deficits are relatively specific to Alzheimer’s disease and unrelated to deficits in other cognitive domains—such as attention or episodic memory—that might underlie the symptomatology of MCI− patients. Secondary outcome measures suggested that MCI+ patients are specifically impaired in distance estimation, as evidenced by reduced proportional linear errors, in line with previous research (Hort *et al.*, 2007), and may relate to tau-related disruption of grid cell activity (Fu *et al.*, 2017; Stangl *et al.*, 2018), given the role of grid cells in computing a distance metric of an environment (Bush *et al.*, 2015) as part of path integration (McNaughton *et al.*, 2006).

No group differences in performance errors were observed in response to the removal of boundary or surface detail cues. In the MCI+ group, a trend toward increased proportional angular errors in response to the removal of boundary (*P = *0.02) and textural (*P = *0.08) cues was observed, but this did not survive multiple comparison correction. Given that this effect did not reach corrected statistical significance, any inferences need to be made with caution. Nonetheless it is worth noting that this trend is consistent with previous research that reported heightened increased reliance on landmark cues (Kalová *et al.*, 2005) and heightened rotational deficits in response to the disruption of optic flow (Kavcic *et al.*, 2006; Mapstone *et al.*, 2008).

Decreased volume was observed in the MCI group compared to controls in regions of interest chosen for their role in path integration (total EC; including partial pmEC and alEC subdivisions, hippocampus, isthmus and posterior cingulate cortex), although only significant differences in hippocampal, total EC, pmEC and alEC volumes survived correction for planned comparisons. In contrast to previous research (Dickerson and Wolk, 2013; Long *et al.*, 2018) no difference in region of interest volumetry across MCI+ and MCI− patients survived correction, though this observation may be influenced by the sample sizes of these two groups. However, consistent with our hypothesis, total EC and partial pmEC subdivision volumes were negatively associated with absolute distance errors across all participants, contrasting with the lack of association between this behavioural measure and alEC, hippocampal, PCC and isthmus volume. Additional analyses demonstrate that both EC and partial pmEC volumes are better predictors of absolute distance errors than either hippocampal or alEC volumes. These findings reinforce previous work suggesting that the EC is critically involved in path integration, and that path integration is more dependent on the EC, and specifically the pmEC subdivision, than on the hippocampus or retrosplenial cortex (Shrager *et al.*, 2008; Kim *et al.*, 2013). To our knowledge, this is the first demonstration that reduced pmEC volumes are associated with impaired path integration in humans, and is consistent with the analogous role of the rodent mEC in path integration (McNaughton *et al.*, 2006; Knierim *et al.*, 2014). These findings complement previous research that demonstrates a relationship between the structure and function of the alEC in ageing and individuals at higher risk of Alzheimer’s disease (Olsen *et al.*, 2017; Berron *et al.*, 2018) and sheds further light on the functional differentiation of the EC (Maass *et al.*, 2015).

Significant negative associations were observed between absolute distance errors and both total EC and pmEC volumes, but not hippocampal volume. While these findings may support the role of the EC, and specifically pmEC, in path integration above and beyond the hippocampus, this interpretation must be applied with caution given that the significant structure-function association was only observed across all participants and not solely within the MCI+ group predicted to have EC degeneration. Future studies with increased sample size will be needed to explore this further.

Path integration performance differentiated the total MCI patient group from healthy control subjects with moderate classification accuracy (AUC 0.82), reflecting the large variance in performance within the former group. By comparison, path integration performance was highly sensitive and specific for prodromal Alzheimer’s disease, classifying this group with an accuracy (AUC 0.90) that was markedly higher than that of reference cognitive tests of episodic memory, attention and processing speed widely used to diagnose prodromal Alzheimer’s disease and as outcome measures in clinical trial.

This work contributes to the growing body of evidence that spatial behavioural tests may have added value, above and beyond traditional cognitive tests, in detecting pre-dementia Alzheimer’s disease (Moodley *et al.*, 2015; Allison *et al.*, 2016; Coughlan *et al.*, 2018; Ritchie *et al.*, 2018). Furthermore, it demonstrates the potential added diagnostic value of a test based around theories of EC function. While this study focused on navigation, given knowledge of the underlying neural basis and the associated translational benefits of such an approach, there is evidence that the transentorhinal and alEC may be affected earlier than the pmEC by the spread of tau (Braak and Braak, 1991; Khan *et al.*, 2014). As such, ongoing work involves extension of VR testing to encompass paradigms relating to object memory, reflecting transentorhinal and alEC function (Deshmukh and Knierim, 2011; Olsen *et al.*, 2017; Berron *et al.*, 2018).

One possible pathological explanation for these data is that impaired path integration in patients with prodromal Alzheimer’s disease is due to spread of tau from the transentorhinal cortex to the pmEC and hippocampus and/or accumulation of amyloid-β pathology in the retrosplenial cortex. The notion that the behavioural impairments are related to Alzheimer’s disease pathology is reinforced by the observation of a significant association between path integration performance and both CSF total tau and CSF amyloid-β. The notion that the behavioural impairments are related to Alzheimer’s disease pathology is reinforced by the observation of a significant association between path integration performance and both CSF total tau and CSF amyloid-β. Given converging data from studies on the initial cortical distribution of Alzheimer’s disease molecular pathology (Braak and Braak, 1991; Bejanin *et al.*, 2017; Whitwell *et al.*, 2018), with tau deposition in the EC and amyloid-β in the retrosplenial cortex, the path integration deficits observed here in prodromal Alzheimer’s disease may relate to a combination of tau and amyloid-β-related dysfunction in the EC and retrosplenial cortex, respectively. Future studies using amyloid- and tau-PET will investigate these potential associations between molecular pathology and navigational behaviour in the early stages of Alzheimer’s disease further, and the relative contribution of amyloid-β and tau to the observed behavioural deficits.

This study has limitations. The sample size of both MCI+ and MCI− groups was relatively small, and these results therefore need to be considered initial findings that require replication in larger scale studies. Another limitation concerns the test space available with the commercial iVR hardware. The use of a larger space, which will be possible with next generation iVR, would likely result in the (i) exclusion of fewer trials; (ii) evaluation of proportional linear errors that is not skewed towards an undershoot; and (iii) the compounding of vector computation errors (angular and linear estimates) that would likely culminate in larger between group performance differences. Finally, the lack of an anatomical mask for automated measurement of the retrosplenial cortex, necessitating the use of proximal anatomical measures (PCC and isthmus cingulate cortex volumes), limits the specificity of analysis of the possible contribution of retrosplenial cortex dysfunction to the path integration impairment in prodromal Alzheimer’s disease.

In conclusion, this study demonstrates that performance on an EC-based iVR path integration task is sensitive and specific for prodromal Alzheimer’s disease, with greater classification accuracy than that of a battery of current ‘gold standard’ cognitive tests. Given that this test is based on understanding of EC grid cell activity, these findings have implications not just for early diagnosis but also for translational Alzheimer’s disease research aimed at understanding mechanistic links between impaired cell activity and behaviour in Alzheimer’s disease. The task used in this study, combined with analogous navigation tasks in animal models of Alzheimer’s disease, would help address the need for outcome measures capable of comparing treatment effects across preclinical and clinical phases of future treatment trials aimed at delaying or preventing the onset of dementia.

## Supplementary Material

awz116_Supplementary_Material

## Abbreviations

4MT: Four Mountains Task
ACE-R: Addenbrookes Cognitive Examination-Revised
al/m/pmEC: anterior-lateral/medial/posteromedial entorhinal cortex
AUC: area under the curve
iVR: immersive virtual reality
MCI(+/−): mild cognitive impairment (with/without CSF evidence of underlying Alzheimer’s disease)
PCC: posterior cingulate cortex
